# Association of blood total immunoglobulin E and eosinophils with radiological features of bronchiectasis

**DOI:** 10.1186/s12890-023-02607-0

**Published:** 2023-08-31

**Authors:** Jiaqi Ren, Ai Chen, Jun Wang, Chun Chang, Juan Wang, Lina Sun, Yongchang Sun

**Affiliations:** Department of Respiratory and Critical Care Medicine, Research Center for Chronic Airway Diseases, Peking University Third Hospital, Peking University Health Science Center, Beijing, China

**Keywords:** Bronchiectasis, Total IgE, Eosinophil, Radiological

## Abstract

**Background:**

Our study aimed to investigate whether serum total IgE and blood eosinophils were associated with radiological features of bronchiectasis in a Chinese cohort.

**Methods:**

We retrospectively enrolled bronchiectasis patients who visited Peking University Third Hospital from Jan 1^st^, 2012 to Oct 7^th^, 2021. The clinical, laboratory and chest CT characteristics were analyzed in association with serum total IgE level and blood eosinophil count.

**Results:**

A total of 125 bronchiectasis patients were enrolled, with 50.4% (63/125) female, and a mean age of 62.4 ± 14.1 years. The median serum total IgE level and blood eosinophil count were 47.7 (19.8, 123.0) KU/L and 140 (90, 230) cells/µl, respectively. In patients with a higher than normal (normal range, 0–60 KU/L) total IgE (43.2%, n = 54), more lobes were involved [4 (3, 5) vs. 3 (2, 4), p = 0.008], and mucus plugs were more common (25.9% vs. 9.9%, p =0.017) on HRCT, as compared to those with a normal level of total IgE. The higher IgE group was more likely to have bilateral involvement (p = 0.059), and had numerically higher Smith and Bhalla scores, but the differences were not statistically significant. In patients with an eosinophil count ≥ 150 cells/µl (49.6%, n = 62), the number of lobes involved was greater [4 (3, 5) vs. 3 (2, 4), p = 0.015], and the Smith and Bhalla scores were higher [9 (5, 12) vs. 6 (3, 9), p = 0.009, 7 (5, 11) vs. 5 (3, 9), p = 0.036]. The Smith score was correlated positively with the eosinophil count (r = 0.207, p = 0.020). Fractional exhaled nitric oxide (FeNO) was correlated with total IgE (r = 0.404, p = 0.001) and eosinophil count (r = 0.310, p = 0.014).

**Conclusions:**

Our study demonstrated that serum total IgE and the blood eosinophil count were associated with the radiological extent and severity of bronchiectasis, necessitating further investigation on the role of T2 inflammation in structural abnormalities of this heterogeneous disease.

**Supplementary Information:**

The online version contains supplementary material available at 10.1186/s12890-023-02607-0.

## Introduction

Bronchiectasis is defined as abnormal dilation of the bronchi, which typically presents with chronic respiratory symptoms including cough, sputum production, and hemoptysis, resulting in impaired quality of life and lung function decline [1]. The incidence, prevalence and disease burden of bronchiectasis are increasing worldwide [2, 3].

Although bronchiectasis is characterized by neutrophil-predominant airway inflammation, emerging evidence suggests that inflammation in bronchiectasis is heterogeneous, with a subpopulation of patients showing features of type 2 inflammatory response, as demonstrated by higher eosinophil counts and percentages in blood [4–7], sputum [4, 8, 9] and bronchial biopsies of airways [10]. Recent studies showed that bronchiectasis with higher blood eosinophil counts was a different phenotype in terms of clinical manifestation, lung function and mortality [4,5,11].

Blood immunoglobulin E (IgE) was also found to be higher in some bronchiectasis patients without allergic bronchopulmonary aspergillosis (ABPA) [12]. One study found that 37% of the bronchiectasis patients had a total IgE level higher than 100 IU/L, and the total IgE level was higher in patients with more severe disease [13].More interestingly, a high frequency of sensitization to multiple allergens was also demonstrated in bronchiectasis patients, and this immuno-allergic subtype was associated with poorer clinical outcomes including decreased pulmonary function and more severe disease [14].

Our previous study [15] found that an increased level of serum total IgE was a risk factor for coexistent bronchiectasis, and correlated positively with the extent of bronchiectasis in patients with chronic obstructive pulmonary disease (COPD). However, no study has investigated the relationship between serum total IgE level and the radiological features of bronchiectasis in patients with non-cystic fibrosis bronchiectasis. Therefore, the current study aimed to investigate whether serum total IgE level and blood eosinophil count, both T2 inflammation biomarkers, were associated with chest HRCT features of bronchiectasis, providing evidence for further investigations on the role of T2 inflammation in airway destruction of this heterogeneous disease.

## Methods

### Patients and data collection

We retrospectively enrolled bronchiectasis patients who visited Peking University Third Hospital from Jan 1st, 2012 to Oct 7th, 2021. The inclusion criteria were as follows: (1) diagnosis fulfilled the 2021 expert consensus on the diagnosis and treatment of adult bronchiectasis in China [16]; (2) age ≥ 18 years; (3) chest high-resolution computed tomography (HRCT), blood routine test, serum total IgE, and specific IgE to *Aspergillus fumigatus* were available; and (4) lung function tests were performed within 6 months before or after the visit. The exclusion criteria were as follows: (1) patients who had a diagnosis of asthma based on the referred history of the patients or fullfilled the criteria of ABPA [17]; (2) pregnancy; (3) on chemotherapy for malignancy; and (4) congenital or acquired immunodeficiency (Supplementary Fig. 1). The study and the exemption from informed consent were both approved by the Clinical Research Ethics Committees of Peking University Third Hospital (M2021428). Patient data confidentiality was in compliance with the Declaration of Helsinki. The bronchiectasis cases were identified and reviewed from the electronic medical record system of the hospital. The clinical data included demographics, smoking history, the number of cigarettes smoked, and respiratory symptoms (cough, sputum, hemoptysis, dyspnea), and the number of exacerbations in the previous year [18]. The severity of bronchiectasis was evaluated based on the BSI score [19] and the E-FACED score [20]. The laboratory data included white blood cell count (WBC) and differentials, hemoglobin (HB), serum total IgE, and specific IgE to *Aspergillus fumigatus*. The normal range of total IgE was 0–60 KU/L (Phadia, Thermo Fisher Scientific, Uppsala, Sweden), and a positive specific IgE to *Aspergillus fumigatus* was defined as more than 0.35 KU/L (Phadia, Thermo Fisher Scientific, Uppsala, Sweden). Spontaneous sputum samples were obtained in the early morning and sent for microbiology test within 2 h. A positive sputum culture was defined as the isolation of pathogenic bacteria while contamination was excluded.

Lung function was measured post-bronchodilator (after reversibility test) as forced expiratory volume in first second in percent predicted values (FEV_1_% pred), FEV_1_/forced vital capacity (FEV_1_/FVC), and residual volume/total lung volume (RV/TLC). In a subpopulation of patients, fractional exhaled nitric oxide (FeNO) was measured (NIOX VERO®, CIRCASSIA, Uppsala, Sweden). The patients avoided active or passive smoking and strenuous exercise 12 h before the test, and avoided caffeinated drinks and nitrogen rich food 2 h before the test. FeNO was performed before lung function test and 12 h before blood sample procurement.

### Evaluation of chest HRCT

For the evaluation of bronchiectasis, the Fleischner Society’s criteria [21] were used, and traction bronchiectasis related to pulmonary fibrosis was excluded. The degree of bronchiectasis in each lobe was scored according to the scoring system proposed by Smith [22]: no bronchiectasis was scored 0, 25% scored 1, 25-49% scored 2, 50-74% scored 3, and above 75% scored 4, with a total score ranging from 0 to 24. Patients with a score of 1 were considered normal because mild bronchiectasis in only one lobe may be seen in a significant proportion of healthy people. The severity of bronchiectasis in each lobe was graded by the Bhalla score [23]: 0, no involvement; 1, mild, luminal diameter slightly greater than diameter of adjacent blood vessel; 2, moderate, lumen 2–3 times the diameter of adjacent vessel; and 3, severe, lumen > 3 times the diameter of adjacent vessel, resulting in a total score ranged from 0 to 18. According to the morphological characteristics, bronchiectasis was classified into three types: cylindrical, cystic or mixed (both cylindrical and cystic). A mucus plug was identified as areas of opacification within the airway lumen with less radio-density than adjacent blood vessels [24]. Two pulmonary physicians evaluated the CT scan for bronchiectasis without knowing the patient’s clinical data. They independently completed the assessment, and differences in readings were resolved through their final consensus.

### Statistical analysis

All data were analyzed using SPSS (version 26.0, IBM, USA). Normally distributed data were presented as mean ± SD and compared by Student’s t test. Data not distributed normally were expressed as median (interquartile range, IQR), and differences were tested by the Mann–Whitney U test. The chi-square test was used to compare categorical data and percentages between groups. The Spearman correlation method was used for the correlation test. A P value < 0.05 was considered to be statistically significant.

## Results

### Demographics and clinical characteristics of the patients

A total of 125 patients diagnosed with bronchiectasis were included for analysis in the study. As shown in Table 1, there was a comparable number of males (63, 50.4%) and females (62, 49.6%), with a mean age of 62.37 ± 14.11 years. Former or current smokers accounted for 25.44% (36/124) of the patients.

**Table 1:** Demographics and clinical characteristics of the patients

	Total (n = 125)
Male/Female	62/63
Age (mean ± SD, years)	62.37 ± 14.11
BMI (mean ± SD, kg/m2)	22.80 ± 3.136
Ever-smoker (n, %)	36/124, 25.44%
Smoking Index (median, IQR)	560 (270, 800)
Respiratory symptoms (n, %)	
Cough	120/125, 96.0%
Sputum	118/125, 94.4%
Hemoptysis	42/125, 33.6%
Dyspnea	55/125, 44.0%
Annual exacerbations (median, IQR)	1.0 (1.0, 2.0)
Blood test	
WBC (10^9^/l, median, IQR)	6.40 (5.13, 7.84)
HB (g/l, median, IQR)	132.3 ± 18.07
Neutrophil (10^9^/l, median, IQR)	4.01 (3.00, 5.02)
Eosinophil (%, median, IQR)	2.20 (1.40, 3.60)
Eosinophil (cell/ul, median, IQR)	140 (90, 230)
Total IgE (KU/L, median, IQR)	47.7 (19.8, 123.0)
Specific IgE to A*spergillus fumigatus* (n, %)	3/125, 2.4%
Sputum culture	89/125, 72.2%
Isolation of any pathogenic bacteria	22/89, 24.7%
Isolation of *Pseudomonas aeruginosa*	15/22, 68.2%
Chest HRCT	
Number of lobes involved (median, IQR)	4 (2, 5)
≥ 3 lobes involved (n, %)	87/125, 69.6%
Bilateral involvement(n, %)	86/125, 68.8%
Lobes involved (n, %)	
Upper lobes	76/125, 60.8%
Middle/ lingula lobe	103/125, 82.4%
Lower lobes	117/125, 93.6%
Smith score(median, IQR)	7 (4, 11)
Bhalla score(median, IQR)	6 (3, 10)
Bronchiectasis type (n, %)	
Cylindrical	38/125, 30.4%
Cystic and/or mixed	87/125, 69.6%
Mucus plugs	21/125, 16.8%
Lung function (n = 125)	
FEV_1_% predicted (%, median, IQR)	73.0 (49.4, 86.7)
FEV_1_/FVC (%, median, IQR)	71.0 (56.7, 77.3)
RV/TLC (mean ± SD, years)	50.3 ± 12.92
Treatment	
ICS + LABA	10/125, 8%
LABA + LAMA	2/125, 1.6%
Long-term(> 2 wk)macrolides*	3/125, 2.4%
BSI score (median, IQR)	9.0 (7.0, 13.0)
0–4	16/89, 18.0%
5–8	25/89, 28.1%
≥9	48/89, 53.9%
E-FACED score (median, IQR)	2.0 (1.0, 4.0)
0–3	53/89, 59.6%
4–6	31/89, 34.8%
7–9	5/89, 5.6%

BMI: body mass index; WBC: white blood count; HB: hemoglobin; HRCT: high resolution computerized tomography; FEV_1_: forced expiratory volume in first second; FVC: forced vital capacity; RV: residual volume; TLC: total lung capacity; ICS: inhaled corticosteroids; LABA: long-acting beta- agonists; LAMA: long-acting muscarinic antagonists; BSI: bronchiectasis severity index; E-FACED: exacerbations, FEV_1_% pred, age, chronic colonisation by *Pseudomonas aeruginosa*, radiological extension and dyspnea

**Fig. 1:**
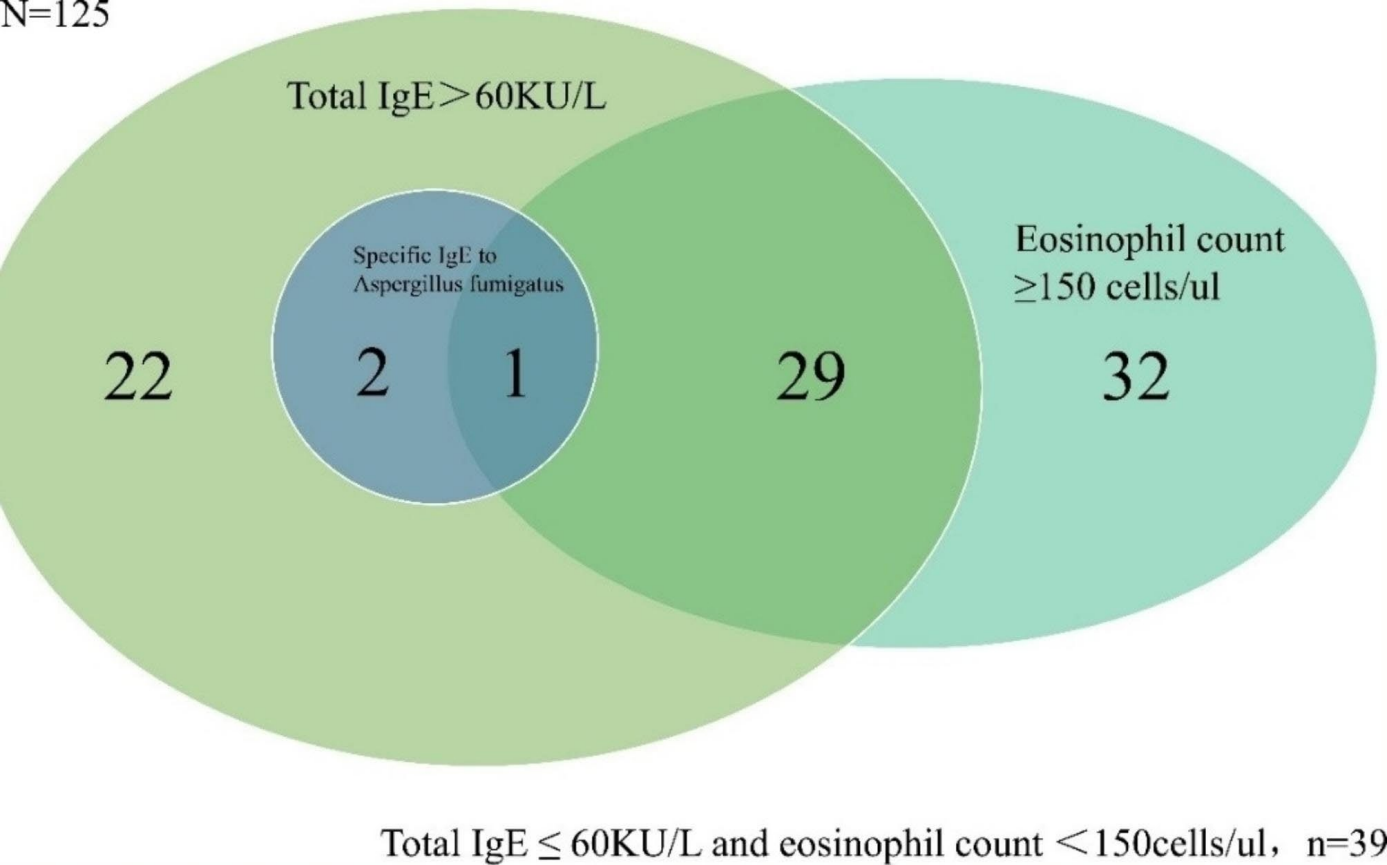
Venn Diagram of serum Total IgE, blood eosinophil count and specific IgE to Aspergillus fumigatus in all the patients

The symptom of cough, sputum production, hemoptysis, and dyspnea was reported in 96.0%, 94.4%, 33.6%, and 44.0% of the patients, respectively. Annual exacerbations of bronchiectasis in the past year were 1.0 (1.0, 2.0). The median percentage and count of blood eosinophils were 2.20 (1.40, 3.60) % and 140 (90, 230) cells/µl, respectively. The median serum total IgE level was 47.7 (19.8, 123.0) KU/L, and 43.2% (54/125) of the patients had a higher than normal level of total IgE (normal range, 0-60KU/L). However, the percentage of patients who had a positive specific IgE to *Aspergillus fumigatus* was low, 2.4% (3/125) in all patients and 5.6% (3/54) in those with a higher total IgE, as shown in Fig. 1.

Regarding the radiological features of bronchiectasis on HRCT, 87.2% (109/125) of patients had involvement of at least two lobes, with a median number of lobes involved of 4 (2, 5), and 68.8% (86/125) had bilateral disease. The median scores of Smith and Bhalla were 7 (4, 11) and 6 (3, 10), respectively. The bronchiectasis was cylindrical in 30.4% (38/125) of the patients, and cystic or mixed in 69.6% (87/125). Mucus plugs were found in 16.8% (21/125) of the patients.

Postbronchodilator FEV1%pred, FEV1/FVC, and RV/TLC were 73.0 (49.4, 86.7) %, 71.0 (56.7, 77.3) %, and 50.3 ± 12.92%, respectively, with 43.2% (54/125) of the patients having obstructive ventilation dysfunction. The BSI score and the E-FACED score were 9.0 (7.0, 13.0) and 2.0 (1.0, 4.0), respectively.

### Comparison of radiological features of bronchiectasis according to levels of total IgE

We divided the patients into two groups based on the level of total IgE, a higher than normal group (> 60KU/L), and a normal group. In the higher IgE group, the median number of lobe involvement was greater [4 (3, 5) vs. 3 (2, 4), p = 0.008], and the percentage of patients with lobes involved ≥ 3 was higher (81.5% vs. 60.6%, p = 0.012). Bilateral involvement tended to be more common (77.8% vs. 62.0%, p = 0.059) in the higher IgE group, which also showed numerically higher Smith and Bhalla scores, but the differences were not statistically significant. There was no difference in the types (cylindrical or cystic) of bronchiectasis between the two groups, but mucus plugs were more common in the higher IgE group (25.9% vs. 9.9%, p = 0.017), shown in Table 2.

**Table 2:** Demographic, clinical, laboratory and radiological features according to blood total IgE level

	Total IgE ≤ 60 (KU/L)	Total IgE>60(KU/L)	P value
No. Patients (total n = 125)	n = 71, 56.8%	n = 54, 43.2%	
Male/Female	38/33	24/30	0.315
Age (mean ± SD, years)	61.30 ± 15.37	63.78 ± 12.25	0.317
BMI (mean ± SD, kg/m^2^)	21.88 ± 3.064	23.95 ± 2.854	**0.000**
Ever-smoker (n, %)	18/70, 27.91%	17/54, 22.9%	0.593
Smoking Index (median, IQR)	760 (300, 800)	50 (150, 600)	0.215
Respiratory symptoms (n, %)			
Cough	70/71, 98.6%	50/54, 92.6%	0.090
Sputum	67/71, 94.4%	51/54, 94.4%	0.985
Hemoptysis	25/71, 35.2%	17/54, 31.5%	0.662
Dyspnea	31/71, 43.7%	24/54, 44.4%	0.930
Annual exacerbations (median, IQR)	1.0 (1.0, 2.0)	1.0 (1.0, 2.0)	0.913
Blood test			
WBC (10^9^/l, median, IQR)	6.06 (5.05, 7.59)	6.74 (5.30, 7.96)	0.347
HB (g/l, median, IQR)	132.7 ± 18.61	131.9 ± 17.48	0.799
Neutrophil (10^9^/l, median, IQR)	3.77 (2.98, 4.78)	4.24 (3.07, 5.38)	0.300
Eosinophil (%, median, IQR)	2.20 (1.40, 3.20)	2.50 (1.40, 5.20)	0.205
Eosinophil (cell/ul, median, IQR)	130 (80, 210)	170 (100, 300)	0.137
Positive specific IgE to *Aspergillus fumigatus* (n, %)	0/71, 0%	3/54, 5.6%	**0.044**
Sputum culture			
Negative	38/53, 71.7%	29/36, 80.6%	0.654
Isolation of *Pseudomonas aeruginosa*	10/53, 18.9%	5/36, 13.9%	
Isolation of other bacteria^§^	5/53, 9.4%	2/36, 5.6%	
Chest HRCT			
Number of lobes involved (median, IQR)	3 (2, 4)	4 (3, 5)	**0.008**
≥ 3 lobes involved (n, %)	43/71, 60.6%	44/54, 81.5%	**0.012**
Bilateral involved (n, %)	44/71, 62.0%	42/54, 77.8%	0.059
Lobes involved (n, %)			
Upper lobes	40/71, 56.3%	36/54, 66.7%	0.241
Middle/ lingula lobe	57/71, 80.3%	46/54, 85.2%	0.476
Lower lobes	65/71, 91.5%	52/54, 96.3%	0.283
Smith score (median, IQR)	6 (3, 11)	7 (4, 11)	0.552
Bhalla score (median, IQR)	6 (3, 9)	7 (4, 10)	0.128
Bronchiectasis type (n, %)			
Cylindrical	21/71, 29.6%	17/54, 31.5%	0.819
Cystic and/or mixed	50/71, 70.4%	37/54, 68.5%	
Mucus plugs	7/71, 9.9%	14/54, 25.9%	**0.017**
Lung function			
FEV_1_% predicted (%, median, IQR)	68.0 (40.4, 83.0)	79.0 (64.0, 93.5)	**0.017**
FEV_1_/FVC (%, median, IQR)	67.3 (51.0, 77.0)	71.9 (66.0, 79.0)	0.149
RV/TLC (mean ± SD, years)	50.3 ± 13.44	50.2 ± 12.32	0.965
BSI score (median, IQR)	9.0 (6.5, 13.0)	9.0 (8.0, 12.8)	0.983
0–4	10/53, 18.9%	6/36, 16.7%	0.903
5–8	14/53, 26.4%	11/36, 30.6%	
≥9	29/53, 54.7%	19/36, 52.8%	
E-FACED score (median, IQR)	3.0 (1.0, 4.0)	2.0 (2.0, 4.0)	0.889
0–3	32/53, 60.4%	21/36, 58.3%	0.978
4–6	18/53, 34.0%	13/36, 36.1%	
7–9	3/53, 5.7%	2/36,5.6%	

BMI: Body Mass Index; WBC: white blood count; HB: hemoglobin; HRCT: high resolution computerized tomography; FEV_1_: forced expiratory volume in first second; FVC: forced vital capacity; RV: residual volume; TLC: total lung capacity; BSI: bronchiectasis severity index; E-FACED: exacerbations, FEV1% pred, age, chronic colonisation by Pseudomonas aeruginosa, radiological extension and dyspnea.; Bold: P values＜0.05. §: Haemophilus influenza, Klebsiella pneumonia, Klebsiella ozaenae, etc.

### Comparison of radiological features of bronchiectasis according to blood eosinophil counts

**Fig. 2:**
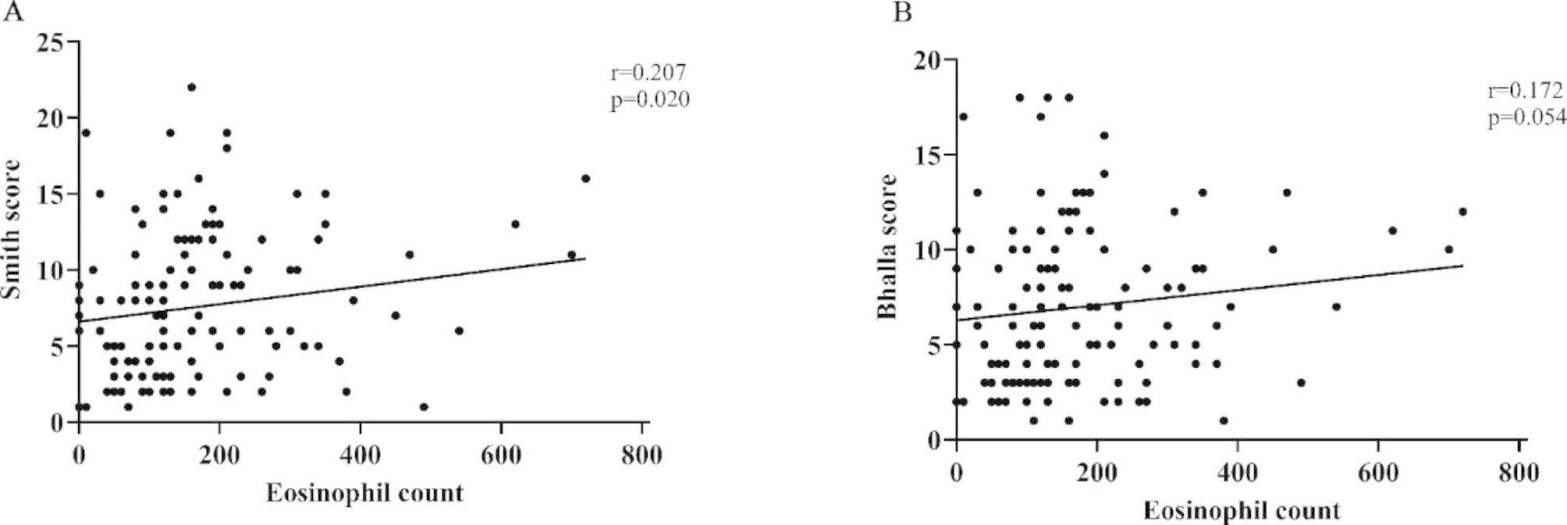
Relationship between eosinophil count and radiological features of bronchiectasis (**A**. Smith score; **B**. Bhalla score)

The patients were then divided into two groups based on the blood eosinophil count: those ≥ 150 cells/µl and < 150 cells/µl, which resulted in an approximately equal number of patients in each group (49.6% vs. 50.4%). In the higher eosinophil group, the number of lobes involved was greater [4 (3, 5) vs. 3 (2, 4), p = 0.015], and both the Smith score [9 (5, 12) vs. 6 (3, 9), p = 0.009] and the Bhalla score [7 (5, 11) vs. 5 (3, 9), p = 0.036], were higher, as shown in Table 3. We also grouped the patients by the cut-off value of blood eosinophil count as 300 cells/uL, and the results showed a similar trend (see Supplementary Table 1). It was interesting to note that the eosinophil count was correlated positively, although weakly, to the Smith score (r = 0.207, p = 0.020), and the Bhalla score, (r = 0.174, p = 0.054), as shown in Fig. 2.

**Table 3:** Demographic, clinical, laboratory and radiological features according to blood eosinophil counts

	Eosinophil<150 cell/ul	Eosinophil ≥ 150 cell/ul	P value
No. patients (total n = 125)	n = 63, 50.4%	n = 62, 49.6%	
Male/Female	1:1.25 (28:35)	1.30:1 (35:27)	0.179
Age (mean ± SD, years)	62.44 ± 14.269	62.29 ± 14.06	0.952
BMI (mean ± SD, kg/m2)	22.62 ± 3.286	22.98 ± 3.00	0.530
Ever-smoker (n, %)	15/62, 24.2%	21/62, 33.9%	0.301
Smoking Index (median, IQR)	600 (240, 800)	500 (300, 760)	0.359
Respiratory symptoms (n, %)			
Cough	61/63, 96.8%	59/62, 95.2%	0.635
Sputum	62/63, 98.4%	56/62, 90.3%	**0.049**
Hemoptysis	23/63, 36.5%	19/62, 30.6%	0.488
Dyspnea	23/63, 36.5%	32/62, 51.6%	0.089
Annual exacerbations (median, IQR)	1.0 (1.0, 2.0)	1.0 (1.0, 2.0)	0.108
Blood test			
WBC (10^9^/l, median, IQR)	6.40 (4.94, 7.89)	6.47 (5.33, 7.84)	0.799
HB (g/l, median, IQR)	132.0 ± 16.12	132.7 ± 19.98	0.831
Neutrophil (10^9^/l, median, IQR)	4.01 (3.01, 5.06)	4.02 (2.98, 5.00)	0.855
Total IgE (KU/L, median, IQR)	40.7 (16.5, 106.0)	57.9 (22.6, 153.0)	0.325
Total IgE > 60KU/L (n, %)	24/63, 38.1%	30/62, 48.4%	0.245
Positive specific IgE to *Aspergillus fumigatus* (n, %)	2/62, 3.2%	1/62, 1.6%	0.568
Sputum culture			
Negative	35/47, 74.5%	32/42, 76.2%	0.760
Isolation of *Pseudomonas aeruginosa*	9/47, 19.1%	6/42, 14.3%	
Isolation of other bacteria^§^	3/47, 6.4%	4/42, 9.5%	
Chest HRCT			
Number of lobes involved (n, %)	3 (2, 4)	4 (3, 5)	**0.015**
≥ 3 lobes involved (n, %)	39/63, 61.9%	48/62, 77.4%	0.059
Bilateral involvement (n, %)	39/63, 61.9%	47/62, 75.8%	0.093
Lobes involved (n, %)			
Upper lobes	35/63, 55.6%	41/62, 66.1%	0.226
Middle/ lingula lobe	48/63, 76.2%	55/62, 88.7%	0.066
Lower lobes	58/63, 92.1%	59/62, 95.2%	0.479
Smith score (median, IQR)	6 (3, 9)	9 (5, 12)	**0.009**
Bhalla score (median, IQR)	5 (3, 9)	7 (5, 11)	**0.036**
Bronchiectasis type (n, %)			
Cylindrical	22/63, 34.9%	16/62, 25.8%	0.268
Cystic and/or mixed	41/63, 65.1%	46/62, 74.2%	
Mucus plugs	13/63, 20.6%	8/62, 12.9%	0.248
Lung function			
FEV1% predicted (%, median, IQR)	73.0 (48.0, 85.1)	74.6 (50.6, 87.0)	0.608
FEV1/FVC (%, median, IQR)	71.0 (55.2, 78.5)	71.3 (59.0, 77.2)	0.801
RV/TLC (mean ± SD, years)	50.0 ± 13.87	50.6 ± 11.99	0.807
BSI score (median, IQR)	9.0 (7.0, 13.0)	9.0 (6.8, 12.3)	0.931
0–4	9/47, 19.1%	7/42, 16.7%	0.848
5–8	14/47, 29.8%	11/42, 26.2%	
≥9	24/47, 51.1%	24/42, 57.1%	
E-FACED score (median, IQR)	2.0 (1.0, 4.0)	3.0 (2.0, 4.0)	0.491
0–3	29/47, 61.7%	24/42, 57.1%	0.809
4–6	15/47, 31.9%	16/42, 38.1%	
7–9	3/47, 6.4%	2/42, 4.8%	

BMI: Body Mass Index; WBC: white blood count; HB: hemoglobin; HRCT: high resolution computerized tomography; FEV_1_: forced expiratory volume in first second; FVC: forced vital capacity; RV: residual volume; TLC: total lung capacity; BSI: bronchiectasis severity index; E-FACED: exacerbations FEV_1_% pred, age, chronic colonisation by *Pseudomonas aeruginosa*, radiological extension and dyspnea; Bold: P values<0.05; ^§^: Haemophilus influenza, Klebsiella pneumonia, Klebsiella ozaenae, etc.

### Association of FeNO with blood total IgE and eosinophils

**Fig. 3:**
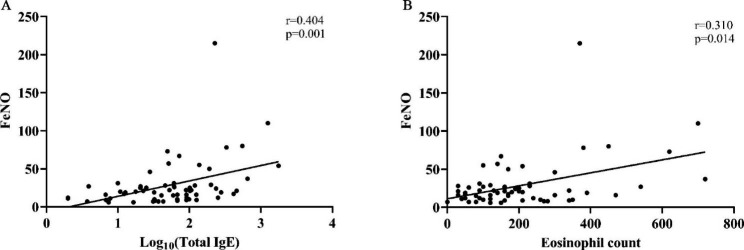
Association of FeNO with Log10 (Total IgE) (**A**) and eosinophil count (**B**)

In 63 bronchiectasis patients who received FeNO measurement, the median level of FeNO was 20 (11, 28) ppb, and 39.7% (25/63) were above 25 ppb. The level of FeNO was well correlated with blood total IgE (r = 0.404, p = 0.001) and eosinophil count (r = 0.310, p = 0.014), as shown in Fig. 3.

## Discussion

In this cohort of 125 patients with bronchiectasis not concurrent with clinical asthma or ABPA, 56.8% had higher than normal levels of serum total IgE, and 49.6% had blood eosinophil counts ≥ 150 cells /ul. Both higher IgE levels and higher eosinophil counts were associated with more extensive or more severe bronchiectasis on HRCT, suggesting that T2 inflammation may play an active part in airway damage and remodeling characteristic of this heterogeneous disease. Consistent with previous studies [8], in our cohort, both the blood eosinophil count and the serum IgE level were correlated positively with FeNO, a surrogate of airway eosinophilic inflammation and of the T2 endotype [25].

Airway inflammation in bronchiectasis has been historically recognized as neutrophilic in nature, however, recent studies also revealed a T2 endotype of the disease, as manifested by higher blood and/or sputum eosinophil counts, increased serum total IgE, higher FeNO, and allergy to various antigens [4, 11, 12]. In recent years, novel biomarkers, such as those related to IL-5, IL-33 [26] and COL4A3 [27] are emerging, but their expressions and potential roles in bronchiectasis still await investigation.

As early as 2008, King et al. reported 4 cases of idiopathic bronchiectasis with elevated serum IgE, all having multi-lobar disease (mean number of lobes involved 3, range 2–4) [12], indicating a role of T2 inflammation in extensive airway destruction in bronchiectasis. Up till now, few studies have examined the potential mechanisms by which T2 inflammation participates in bronchiectasis. ABPA is an extreme example of a cause of bronchiectasis that is primarily eosinophilic. Eosinophils also have been described as key players in *Aspergillus fumigatus* lung infection [28]. It is interesting to note that both Aspergillus sensitization and infection tend to have multi-lobe involvement [29]. Another example is nontuberculous mycobacteria (NTM) infection. Bronchiectasis patients with positive NTM were more likely to have diffusely dilated airways [30]. It was found in a UK study that infection of NTM, especially that of *Mycobacterium avium-intracellular* complex, was associated with T2 immune responses [31].A recent retrospective study showed that *Staphylococcus aureus (S. aureus)* in the lower airway may indicate an allergic predisposition with staphylococcal enterotoxin sensitization and blood eosinophilia in bronchiectasis [32], and another previous study showed that a higher number of affected lobes (OR: 1.53; 95% CI: 1.2–1.95; p < 0.001) was independently associated with chronic bronchial infection due to *S. aureus* in patients with bronchiectasis [33]. The relationship between *Pseudomonas aeruginosa* and T2 inflammation is ambiguous. For example, there was no differences in *Pseudomonas aeruginosa* infection or colonization between the T2 and non-T2 groups in a cohort study from the Spanish Online Bronchiectasis Registry [5]. And the evidence of the relationship between *Pseudomonas aeruginosa* and blood eosinophilia was inconsistent in a European multicohort study [4]. There was no difference in microbiome profiles (mainly bacteriologic features) between the two groups of higher and lower eosinophils or higher and normal IgE groups in our study. Perhaps there may be interesting results when the sample size is expanded in future studies.

It is well known that the basic granules of eosinophils contain major basic protein (MBP), while the matrix contains eosinophil cationic protein (ECP), eosinophil derived neurotoxin (EDN), and eosinophil peroxidase (EPO). ECP and MBP are mediators of potent antibacterial and antiparasitic activities [34]. We speculated that the increase of eosinophils in bronchiectasis is not only a manifestation of allergy, but also a response to infection. In an earlier study by Gaga et al. [10] the number of EG2+ (activated) eosinophils in bronchial mucus of post-infective bronchiectasis patients were increased compared to healthy controls matched for atopic status. In keeping with this, low eosinophil counts (< 100 cells/ul) increased the risk of pneumonia in COPD patients with bronchial colonization, particularly in those receiving treatment with inhaled corticosteroids [35].

We found no significant differences in FEV1% and FEV1/FVC between the two groups of higher and lower eosinophils in our study, which was consistent with some previous reports. The recent study of 5 European cohorts showed no difference in FEV1% between groups according to blood eosinophil counts [4]. However, a multicenter, prospective observational study from Spain showed better lung function in bronchiectasis patients with high blood eosinophil counts [5]. It is also intriguing to note that, patients with elevated serum total IgE levels had a higher FEV1% than those with normal IgE levels in our cohort. There was a well-known association between disease extent in HRCT and decreased FEV1 in subjects with bronchiectasis [22, 36, 37]. We also found this inverse correlation between the Smith score or the Bella score and FEV1% in the present study, as shown in supplementary Fig. 2. Although the underlying pathophysiology that may account for these findings remains obscure, it is possible that T2 inflammation or allergy is not a precipitating factor for lung function decline in bronchiectasis.

There is not enough evidence to clarify T2 inflammation and the disease severity of bronchiectasis. A multi-center cohort study showed that bronchiectasis patients with sensitization to three or more allergens had the highest BSI score [14]; In contrast, the European multicohort study reported that bronchiectasis patients with lower blood eosinophil counts (< 100 cells/ul) had the highest BSI scores (p < 0.0001). There was no significant difference in BSI or E -FACED scores between the T2 and non-T2 groups (both eosinophil counts and IgE) in the present study. It was speculated that the BSI and E-FACED scores involved multiple aspects of evaluating the severity of bronchiectasis, such as, imaging and lung function. The role of T2 inflammation in the pathogenesis of bronchiectasis is complex, being associated with poorer imaging scores and better lung function. These opposing effects may be offset when the BSI and E-FACED scores were evaluated. Therefore, the heterogeneity in impacts of T2 inflammation on severity of bronchiectasis may lead to the different results about the BSI and E-FACED scores in different studies.

However, there were several studies which demonstrated the response to biological treatments in bronchiectasis patients with the T2 endotype. Two real-world studies indicated that IL-5 antagonists were effective in reducing oral corticosteroids and improving respiratory function in patients with coexistent bronchiectasis and severe eosinophilic asthma [38, 39]. In a cross-sectional study, five patients with severe eosinophilic asthma and concomitant bronchiectasis accepted treatment with either mepolizumab or benralizumab which significantly reduced the exacerbation rate [11]. In a German single center retrospective study, patients with clinically significant bronchiectasis with an eosinophilic inflammatory endotype, which failed to respond to standard bronchiectasis treatments, were treated with add-on mepolizumab or benralizumab, resulting in a significant reduction of blood eosinophils as well as a significant improvement in FEV1, symptom burden and quality of life [40]. Therefore, there is an urgent need to perform randomized controlled trials on biological treatments targeting the T2-high endotype in bronchiectasis patients.

One of the strengths of this study was that our work revealed, for the first time to our knowledge, the distribution of blood eosinophil counts and serum total IgE levels in a well-characterized cohort of patients with bronchiectasis excluding clinical asthma and ABPA. But our study also had several limitations. Considering that IgE was not a routine test for bronchiectasis in clinical practice, patients receiving IgE measurement might have clinical indications, such as history of allergy or frequent exacerbations, which might lead to selection bias, although clinical asthma was carefully excluded from our analysis. We did not evaluate the number of airway eosinophils, although a relationship between blood and sputum eosinophil counts in bronchiectasis had been demonstrated in 2 European cohorts [4], and blood eosinophilia is an accepted surrogate of airway eosinophilia in several chronic respiratory diseases [4, 41, 42]. Furthermore, we confirmed the correlation between blood eosinophil counts and serum IgE levels and FeNO in the present study. In our study, the results of sputum bacterial culture were limited, which made it impossible to look at the association between bacteriological data and IgE or eosinophils. Because of the retrospective and cross-sectional nature of the study, the patient’s comorbidities, radioallergosorbent / prick tests, and other T2 inflammation biomarkers, as well as the longitudinal changes of esonophils and IgE levels were not available. Finally, the diagnosis of asthma, one of the exclusion criteria of our cohort, was a clinical diagnosis from the doctors, not confirmed by reversible airway obstruction. In fact, it is difficult to determine whether variability in FEV_1_ in an obstructive disease like bronchiectasis is a marker of asthma (or asthmatic trait) or not.

## Conclusion

In conclusion, our study revealed an association of blood eosinophil counts and total IgE levels with the radiological severity of bronchiectasis, suggesting that T2 inflammation may participate in airway structural destruction in bronchiectasis, which warrants further clinical and experimental investigation.

### Electronic supplementary material

Below is the link to the electronic supplementary material.

Additional Files 1. Flowchart of the enrollment of patients.

Additional Files 2. Relationship between FEV1%pred and radiological feature of bronchiectasis (A. Smith score; B. Bhalla score).

Additional Files 3. Demographic, clinical, laboratory and radiological features according to blood eosinophil counts (300 cell/ul).

## Data Availability

All data generated or analyzed during this study are available from the corresponding author upon reasonable request.

## Abbreviations

ABPA: Allergic bronchopulmonary aspergillosis
COPD: Chronic obstructive pulmonary disease
ECP: Eosinophil cationic protein
EDN: Eosinophil derived neurotoxin
EPO: Eosinophil peroxidase
FEV_1_%pred: Forced expiratory volume in the first second in percent predicted values
FEV_1_/FVC: Forced expiratory volume in the first second/forced vital capacity
FeNO: Fractional exhaled nitric oxide
HB: Hemoglobin
HRCT: High-resolution computed tomography
IgE: Immunoglobulin E
IQR: Interquartile range
MBP: Major basic protein
NTM: Nontuberculous mycobacteria
RV/TLC: Residual volume/total lung volume
WBC: White blood cell
