# Supplementary figures and images for: Association of blood total immunoglobulin E and eosinophils with radiological features of bronchiectasis
Source: BMC Pulm Med. 2023 Aug 31;23:316. doi: 10.1186/s12890-023-02607-0 (PMC10472648; doi:10.1186/s12890-023-02607-0)

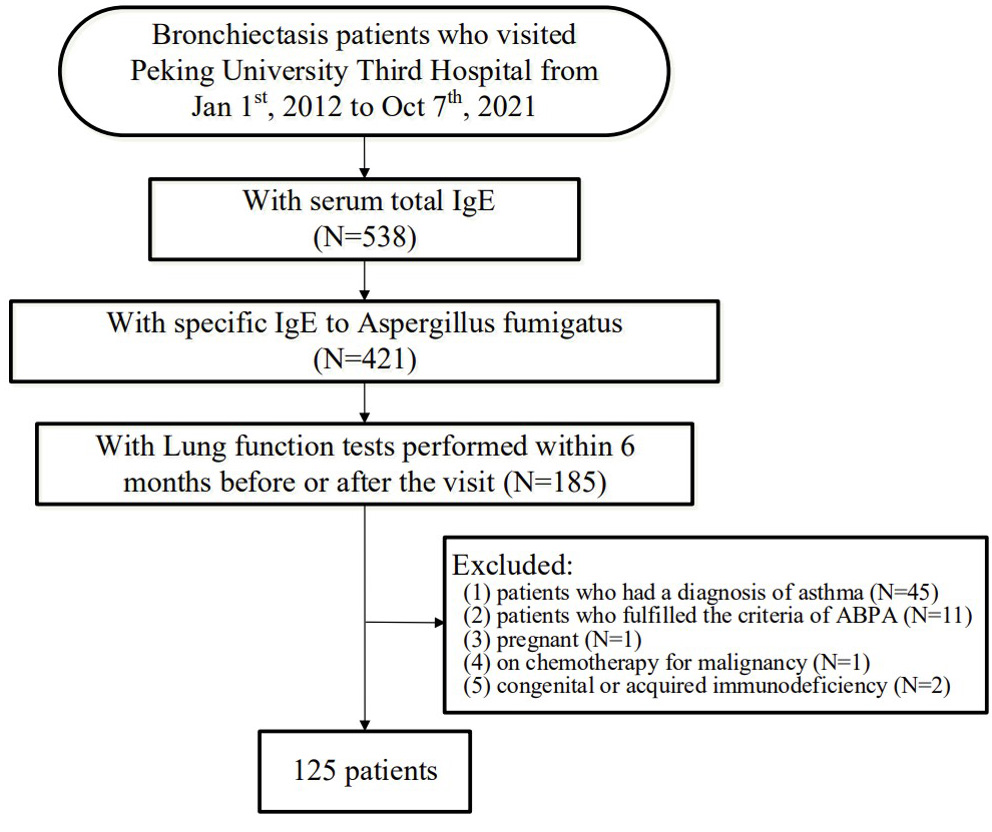


Additional Files 1. Flowchart of the enrollment of patients.

Supplement: Supplementary file 1 — Additional Files 1. Flowchart of the enrollment of patients. [file 12890_2023_2607_MOESM1_ESM.docx]
