# Supplementary material for: Association of blood total immunoglobulin E and eosinophils with radiological features of bronchiectasis
Source: BMC Pulm Med. 2023 Aug 31;23:316. doi: 10.1186/s12890-023-02607-0 (PMC10472648; doi:10.1186/s12890-023-02607-0)
Supplement: Supplementary file 2 — Additional Files 2. Relationship between FEV1%pred and radiological feature of bronchiectasis (A. Smith score; B. Bhalla score). [file 12890_2023_2607_MOESM2_ESM.docx]

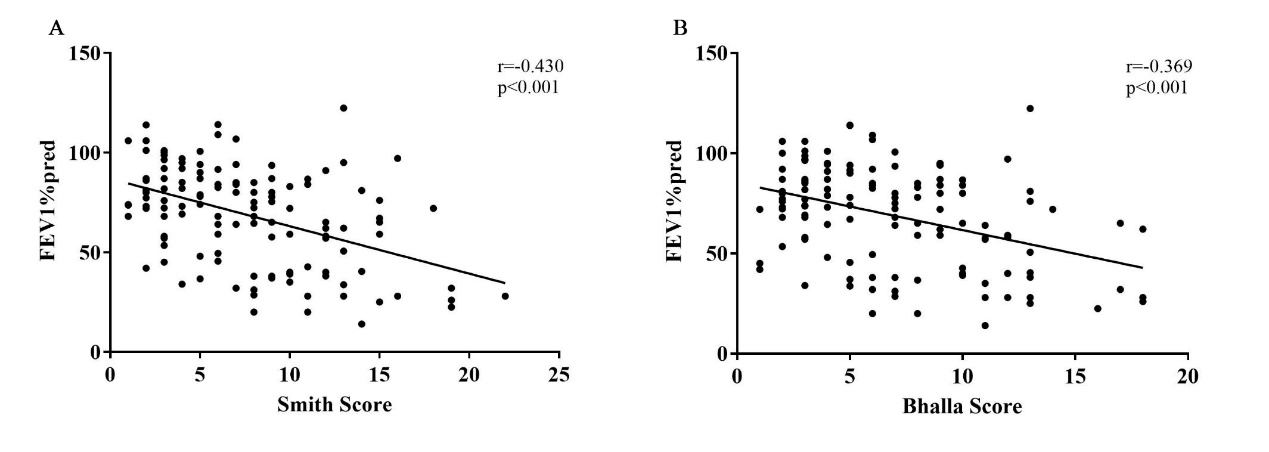


Additional Files 2. Relationship between FEV1%pred and radiological feature of bronchiectasis (A. Smith score; B. Bhalla score).
