# Supplementary material for: Association of blood total immunoglobulin E and eosinophils with radiological features of bronchiectasis
Source: BMC Pulm Med. 2023 Aug 31;23:316. doi: 10.1186/s12890-023-02607-0 (PMC10472648; doi:10.1186/s12890-023-02607-0)
Supplement: Supplementary file 3 — Additional Files 3. Demographic, clinical, laboratory and radiological features according to blood eosinophil counts (300 cell/ul). [file 12890_2023_2607_MOESM3_ESM.docx]

Additional Files 3. Demographic, clinical, laboratory and radiological features according to blood eosinophil counts (300 cell/ul)

|  | Eosinophil＜300 cell/ul | Eosinophil≥300 cell/ul | P value |
| --- | --- | --- | --- |
| No. patients (total n=125) | n=103, 82.4% | n=22, 17.6% |  |
| Male/Female | 1.15:1 (55:48) | 1:2.14 (7:15) | 0.066 |
| Age (mean ± SD, years) | 61.95±14.301 | 64.32±13.318 | 0.477 |
| BMI (mean ± SD, kg/m2) | 22.60±3.131 | 23.70±3.069 | 0.139 |
| Ever-smoker (n, %) | 24/102, 23.5% | 12/22, 54.5% | 0.013 |
| Smoking Index (median, IQR) | 450 (220, 800) | 600 (400, 800) | 0.533 |
| Respiratory symptoms (n, %) |  |  |  |
| Cough | 101/103, 98.1% | 19/22, 86.4% | **0.011** |
| Sputum | 100/103, 97.1% | 18/22, 81.8% | **0.005** |
| Hemoptysis | 39/103, 37.9% | 3/22, 13.6% | **0.029** |
| Dyspnea | 42/103, 40.8% | 13/22, 59.1% | 0.116 |
| Annual exacerbations (median, IQR) | 1.0 (1.0, 2.0) | 1.0 (0.8, 2.0) | 0.505 |
| Blood test |  |  |  |
| WBC (10^9^/l, median, IQR) | 6.35 (5.00, 7.88) | 6.74 (5.44,7 .70) | 0.470 |
| HB (g/l, median, IQR) | 132.1±16.05 | 133.5±25.96 | 0.752 |
| Neutrophil (10^9^/l, median, IQR) | 3.93 (3.00, 5.05) | 4.31 (2.97, 5.02) | 0.920 |
| Total IgE (KU/L, median, IQR) | 40.6 (17.7, 102.0) | 102.3 (32.4, 550.0) | **0.021** |
| Total IgE > 60KU/L (n, %) | 40/103, 38.8% | 14/22, 63.6% | **0.033** |
| Positive specific IgE to *Aspergillus fumigatus* (n, %) | 3/103, 2.9% | 0/22, 0% | 0.418 |
| Sputum culture |  |  |  |
| Negative | 58/76, 76.3% | 9/13, 69.2% | 0.809 |
| Isolation of *Pseudomonas aeruginosa* | 12/76, 14.8% | 3/13, 23.1% |  |
| Isolation of other bacteria^§^ | 6/76, 7.9% | 1/13, 7.7% |  |
| Chest HRCT |  |  |  |
| Number of lobes involved (n, %) | 4 (2, 5) | 4 (3, 6) | 0.116 |
| ≥ 3 lobes involved (n, %) | 70/103, 68.0% | 17/22, 77.3% | 0.389 |
| Bilateral involvement (n, %) | 73/103, 69.9% | 14/22, 63.6% | 0.565 |
| Lobes involved (n, %) |  |  |  |
| Upper lobes | 61/103, 59.2% | 15/22, 68.2% | 0.435 |
| Middle/ lingula lobe | 82/103, 79.6% | 21/22, 95.5% | 0.077 |
| Lower lobes | 97/103, 94.2% | 20/22, 90.9% | 0.570 |
| Smith score (median, IQR) | 7 (3, 10) | 8 (5.0, 12.0) | 0.218 |
| Bhalla score (median, IQR) | 6 (3, 10) | 8 (5.0, 10.0) | 0.144 |
| Bronchiectasis type (n, %) |  |  |  |
| Cylindrical | 16/103, 15.5% | 5/22, 22.7% | 0.413 |
| Cystic and/or mixed | 87/103, 84.5% | 17/22, 77.3% |  |
| Mucus plugs | 18/103, 17.5% | 3/22, 13.6% | 0.662 |
| Lung function |  |  |  |
| FEV1 % predicted (%, median, IQR) | 73.0(45.5, 86.7) | 73.1 (62.0, 90.0) | 0.659 |
| FEV1/FVC (%, median, IQR) | 71.0(55.2, 77.0) | 72.6(64.0, 80.0) | 0.662 |
| RV/TLC (mean ± SD, years) | 50.1±13.33 | 51.0±11.05 | 0.095 |
| BSI score (median, IQR) | 8.0 (5.0, 12.0) | 8.5 (3.0, 13.0) | 0.0.933 |
| 0-4 | 24/103, 23.3% | 8/22, 36.4% | 0.160 |
| 5-8 | 34/103, 33.0% | 3/22, 13.6% |  |
| ≥9 | 45/103, 43.7% | 11/22, 50.0% |  |
| E-FACED score (median, IQR) | 2.0 (1.0, 4.0) | 2.5 (2.0, 4.3) | 0.990 |
| 0-3 | 66/103, 64.1% | 15/22, 68.2% | 0.590 |
| 4-6 | 32/103, 31.1% | 5/22, 22.7% |  |
| 7-9 | 5/103, 4.9% | 2/22, 9.1% |  |

BMI: Body Mass Index; WBC: white blood count; HB: hemoglobin; HRCT: high resolution computerized tomography; FEV1: forced expiratory volume in first second; FVC: forced vital capacity; RV: residual volume; TLC: total lung capacity; BSI: bronchiectasis severity index; E-FACED: exacerbations FEV_1_% pred, age, chronic colonisation by *Pseudomonas aeruginosa*, radiological extension and dyspnea. Bold: P values＜0.05; ^§^: Haemophilus influenza, Klebsiella pneumonia, Klebsiella ozaenae, etc
